# Symphysiodesis with silver-coated plates for the treatment of traumatic symphyseal instability in complex pelvic trauma

**DOI:** 10.1016/j.tcr.2026.101312

**Published:** 2026-03-19

**Authors:** Moritz Riedl, Florian Baumann, Volker Alt

**Affiliations:** a Department of Trauma Surgery, University Hospital Regensburg, Regensburg, Germany

**Keywords:** Fracture related infection, Pelvic trauma, Silver coating, Infection prevention, Polytrauma, Bone infection

## Abstract

**Background:**

Pelvic ring fractures represent a challenging injury pattern, particularly when accompanied by abdominal trauma and soft tissue disruption, increasing the risk of fracture-related infection (FRI). This report highlights the role of silver-coated implants in the staged management of a complex pelvic injury complicated by abdominal infection for prevention of FRI.

**Clinical case:**

A 55-year-old male sustained a bilateral Type C pelvic ring fracture and multiple associated intraabdominal injuries following high-energy trauma. Initial management included C-clamp stabilization, laparotomy, and urogenital reconstruction. Due to severe abdominal soft tissue infection, a staged fixation strategy was applied: minimally invasive posterior ring fixation and temporary anterior external fixation. After abdominal infection control, a xenogenic mesh was used to reconstruct the abdominal wall. Secondary, symphysiodesis was indicated due to persistent instability of the symphysis. The procedure was ultimately performed using autologous bone graft and double plating with silver-coated implants.

**Results:**

Initially, the patient sustained multiple infectious complications, including bacteremia and urogenital infections, requiring prolonged antimicrobial therapy. At long-term follow-up, a stable fusion of the symphysis was achieved without further infection or mechanical complication.

**Conclusion:**

This case demonstrates that silver-coated implants can serve as an effective adjunct in managing pelvic injury patients at high-risk for infection.

## Introduction

Pelvic ring injuries are among the most complex challenges in trauma surgery [1]. This is only due to their complex treatment but also because of the risk of complications, such as vascular and neural injury, soft tissue and visceral damage, and fracture-related infection (FRI). Although the overall incidence of FRI after pelvic fractures is relatively low, such infections can result in severe consequences including multiple revision surgeries, prolonged hospitalization, and impaired functional recovery. The pathogenesis of FRI is multifactorial and influenced by tissue devitalization, vascular compromise, and trauma-induced immunosuppression. In cases involving significant abdominal trauma, the risk of deep infection increases markedly [2].

Given these challenges, selecting an appropriate stabilization strategy is critical. Temporary external fixation and minimally invasive posterior ring stabilization are often preferred in the acute setting to reduce the risk of infection. Definitive fixation of the anterior pelvic ring however requires open reduction and plate fixation. In recent years, silver-coated implants have emerged as a promising tool in infection prevention and treatment due to their antimicrobial properties [3], [4], [5], [6], [7]. This report presents the staged surgical management of a multiply injured patient with pelvic ring instability and secondary abdominal infection, culminating in successful definitive stabilization of the anterior pelvic ring via symphyseal fusion using silver-coated plates.

## Clinical case

**Fig. 1:**
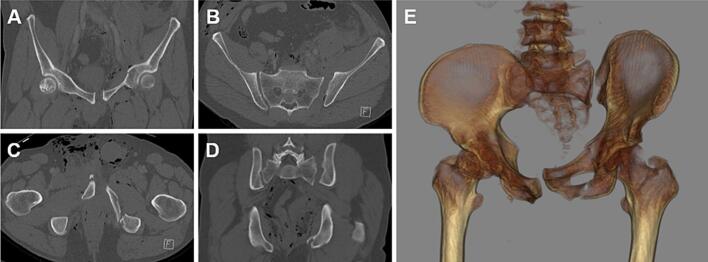
CT scan of the initial pelvic fracture (AO61C2.2) with instability of the symphysis (A, C) and bilateral instability of the posterior pelvic ring in coronal and axial plane (B, D). 3D reconstruction of the initial CT scan (E).

A 55-year-old male sustained severe polytrauma after a motorcycle collision with a car. Injuries included a bilateral Type C pelvic ring fracture (AO61C2.2) with bilateral sacroiliac joint disruption, bilateral fractures of the superior and inferior pubic rami with left acetabular involvement, and symphyseal diastasis (Fig. 1). Additional injuries included severe lumbar soft tissue trauma with tears of the left iliopsoas and paraspinal muscles, disruption of the pelvic floor muscles with traumatic descent of the rectum, bladder, and prostate, bladder rupture, right testicular laceration and dislocation, and lumbosacral plexus injury predominantly affecting the sciatic nerve. An incidental finding revealed a well-differentiated neuroendocrine tumor of the terminal ileum (pT3, L1, V0, pN1 [mi], R0, G1).

Prehospital stabilization included application of a pelvic binder due to mechanical instability. On admission, the patient showed signs of hemorrhagic shock and imaging confirmed the extent of injuries. Initial surgical management by a multidisciplinary team (orthopedic, visceral, and urologic surgeons) included application of a pelvic C-clamp, exploratory laparotomy, bladder and testicular repair, pelvic and abdominal packing, and negative pressure wound therapy. The patient was then admitted to the surgical ICU, requiring high-dose catecholamines and prone positioning for acute respiratory distress syndrome (ARDS).

**Fig. 2:**
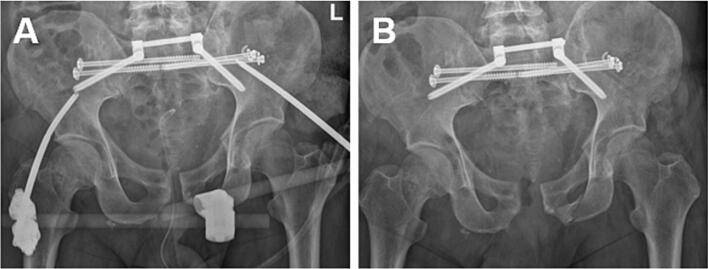
Postoperative anterior-posterior X-ray of the pelvis after posterior stabilization with bilateral sacroiliac screws and transiliac internal fixator and closed reduction and external fixation of the anterior pelvic ring (A). Persistent instability of the symphysis after removal of the external fixator eight weeks post-trauma (B).

A severe abdominal and intrapelvic soft tissue infection developed due to the traumatic urogenital injuries, necessitating prolonged vacuum-assisted wound therapy and multiple surgical revisions. In response, the orthopedic team opted for minimally invasive stabilization of the posterior pelvic ring using bilateral sacroiliac screws and a transiliac internal fixator, combined with external fixation of the anterior ring using a supra-acetabular external fixator (Fig. 2A).

Over the course of intensive care treatment over 30 days, the patient's circulatory status and pulmonary function gradually stabilized, allowing stepwise weaning from ventilatory and vasopressor support.

After stabilization of the soft tissue environment, the abdominal wall was definitively reconstructed at post-injury date 16 with a xenogenic mesh and layered fascial closure.

Eight weeks post-trauma, the anterior external fixator was removed, which showed persisting instability of the symphysis requiring definitive anterior pelvic ring fixation (Fig. 2B).

**Fig. 3:**
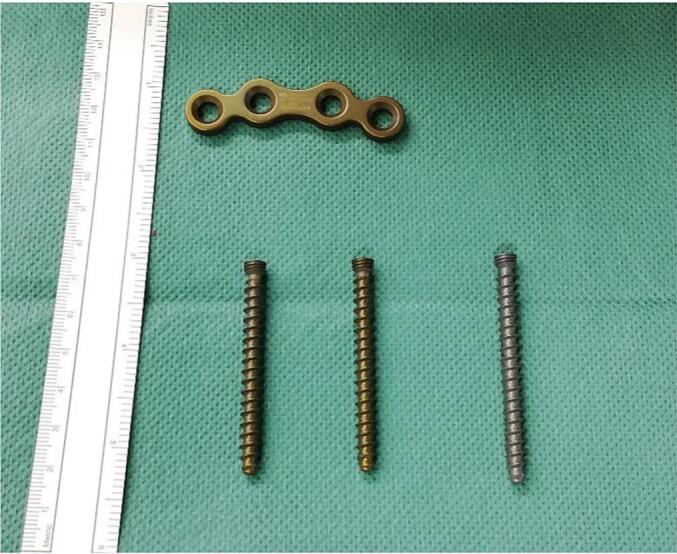
Ultrathin silver plasma-coated (HyProtect®, Bio-Gate AG, Nürnberg, Germany) 4 hole symphysis plate and 5.9 mm locking screws (ITS.®, Graz, Austria) before implantation, which shows a slight colour modification compared to the untreated screw on the right side.

An initial surgical attempt at post-injury day 66 for symphyseal fusion was aborted due to intraoperative signs of infection surrounding the previously implanted abdominal mesh craft. After further debridement and delayed closure, definitive symphyseal fusion was achieved in a second approach using autologous iliac crest bone graft and locking double plating with silver-coated implants (ITS.® symphysis plates with custom-made ultrathin silver plasma coated (HyProtect®, Bio-Gate AG, Nürnberg, Germany)) (Figs. 3 + 4).

**Fig. 4:**
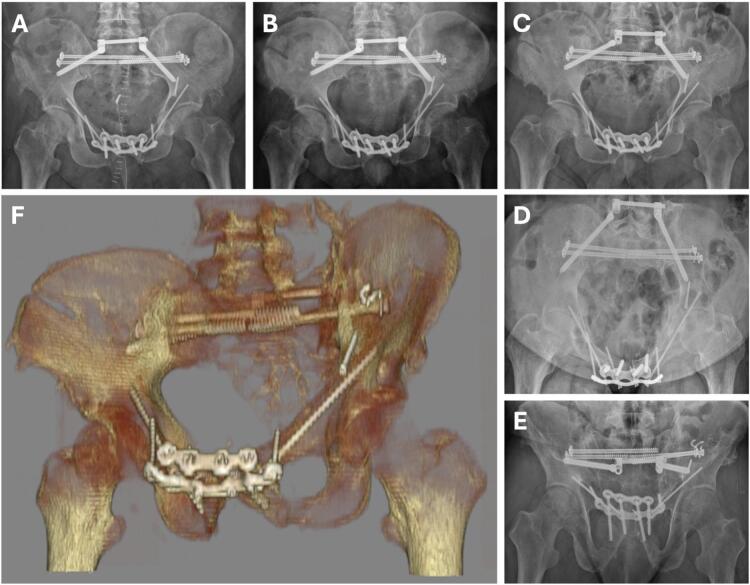
Anterior-posterior X-rays of the pelvis, postoperative after symphysiodesis with custom ultrathin plasma silver coated plates and screws (A), at 6-week follow-up (B), and at 5-year follow-up with loosening of the connector of the transiliac intern fixator including inlet and outlet views (C–E). 3D reconstruction of the final fixation (F).

The incidentally discovered neuroendocrine tumor was managed via segmental resection of the terminal ileum and associated mesentery.

During further hospitalization, the patient experienced multiple infectious complications, including abdominal infection with vancomycin-resistant *Enterococcus faecium* (VRE), bacteremia with VRE and *Candida albicans*, pulmonary HSV-1 reactivation, and urinary tract infections caused by *Citrobacter* spp., *Klebsiella pneumoniae*, and *Staphylococcus haemolyticus* but no FRI of the pelvis. Antimicrobial therapy was adapted to include meropenem, linezolid, caspofungin, and acyclovir. After temporary cessation, a recurrent urinary tract infection required renewed treatment. Ultimately, all infections were successfully resolved, and antimicrobial therapy was discontinued.

Following stabilization, the wounds at the anterior pelvis healed uneventfully and the patient was transferred to a neurological rehabilitation center for management of left-sided sciatic nerve palsy 16 weeks after the trauma and 3 weeks after surgical symphisiodesis. Mobilization was achieved with six weeks of partial weight-bearing (15 kg) on the left leg using forearm crutches.

At long-term follow-up of 5 years, the patient remained infection-free, with a radiologically and clinically stable symphysis without loosening of the silver-coated implants at the anterior pelvic ring. Solely, the right connector of the transiliac internal fixator showed loosening in the radiological follow-up (Fig. 4C). The patient was fully ambulatory without assistive devices. Residual neurological deficits included erectile dysfunction, urge incontinence, and persistent foot drop on the left, managed with a peroneal splint.

## Discussion

This case illustrates the importance of interdisciplinary and staged surgical strategies in managing complex pelvic trauma complicated by soft tissue damage and abdominal infection. The prolonged need for soft tissue treatment necessitated temporizing fixation measures. Ultimately, the use of silver-coated implants enabled the prevention of FRI for the required anterior pelvic ring fixation with sufficient biomechanical stability. This type of surface silver modification offers multiple benefits. Silver exhibits a broad-spectrum antimicrobial effect, effective against both gram-positive bacteria as well as gram-negative organisms and fungi [8]. In contrast to locally applied gentamicin, silver does not carry the risk of promoting antimicrobial resistance. Despite the proven antimicrobial efficacy of silver, potential systemic and local adverse effects, such as argyria, leukopenia, hepatic or renal dysfunction, and local neurotoxicity, must be considered. These events, however, are primarily associated with implants containing high total amounts of silver. The applied coating consists of a siloxane-based ultrathin layer (approximately 10 to 30 nm thick; 1.5 μg/cm^2^), releasing only a low amount of silver ions and demonstrating no cytotoxicity [9], [10]. Further, the use of silver coated implants has been shown not to affect bone healing in experimental studies and several case reports [4], [5], [11]. In this case, no adverse effects associated with silver were observed, and long-term follow-up confirmed the success of this approach.

## Conclusion

Silver-coated implants may offer a valuable adjunct to prevent fracture-related infections at the pelvis for the management of complex pelvic injuries patients at high risk of infection. Their role in staged reconstructions following infection-related complications merits further investigation.

## CRediT authorship contribution statement

**Moritz Riedl:** Writing – original draft, Visualization, Resources, Methodology, Investigation, Formal analysis, Data curation, Conceptualization. **Florian Baumann:** Writing – review & editing, Visualization, Project administration, Data curation, Conceptualization. **Volker Alt:** Writing – review & editing, Supervision, Project administration, Methodology, Investigation, Conceptualization.

## Declaration of competing interest

Volker Alt is a consultant for Bio Gate AG, Nürnberg, Germany. Informed consent for off-label use of silver-coated implants was obtained from the patient.
